# On the Nature and Treatment of Renal Diseases; Being an Inquiry into the Connection of Diabetes, Calculus, and Other Affections of the Kidney and Bladder, with Indigestion

**Published:** 1843-12-30

**Authors:** 


*On the Nature and Treatment of Renal Diseases; being an inquiry into the connection of Diabetes, Calculus, and other affections of the Kidney and Bladder, with Indigestion.* By William Prout, M. D., F. R. S., etc. From the Fourth Revised London Edition, with Plates. Philadelphia: Lea & Blanchard. 1843. 8vo. pp. 465.

The kidney, as the seat and source of disease, has of late years attracted a good deal of attention. The magnificent work of Rayer, in France, together with those of Martin-Solon, and Becquerel, have all appeared within the last five or six years. In England several Pathologists have been actively engaged in the study of the urine; among these, Drs. Willis and Golding Bird have been prominent. To the author of the present work, however, is due the credit of having led the way in the investigation of this class of disorders, and the additions and improvements which each successive edition of his book has contained, show that he is still active in his former pursuits. Besides pursuing the path pointed out by Dr. Prout, all subsequent writers on the subject have largely profited by his labours, and their results have been freely appropriated, too often without any acknowledgement of the obligation. Throughout all the editions, some faultiness and obscurity, both in the style and arrangement, have prevailed, and this has repelled, we fear, too many from consulting these richly stored pages. A diversity of opinion has also existed with regard to the theoretical portions of the volume. Many think that the speculations of the author, although ingenious, have been too hastily formed, and that the present imperfect and vacillating condition of organic Chemistry does not warrant the systematic form he has given to his peculiar doctrines. We have no inclination to enter upon so unprofitable a subject as the discussion of the *questiones vexatæ* of physiology. But granting that his theoretical notions are crude and unsatisfactory, the pages devoted to their exposition are but a small portion of the work. Its great merits, which are positive and distinctive, are of a purely practical character. The exposition and illustration of the intimate relation always existing between a disordered state of the urinary secretion and the condition of the digestive and assimilating functions—a cardinal practical point now recognised by those who are familiar with renal diseases, as of universal application—is the chief feature of the work, and has been the aim and object of the author for years, and to him are we indebted for indicating this all important fact in renal pathology. The study of such a work as this will fully reward any practitioner. The knowledge that he will derive from it will be satisfactory and available; and he will find much for reflexion and future consideration. The more familiar he becomes with it, the less will he regard its imperfections and peculiarities, and the more will he be disposed to acknowledge its great and unrivalled merits—the results of thirty-years of observation and experience.

In the present edition, Dr. Prout has made the introduction, which first appeared in the third edition, the third book. Treating of the General Physiology and Pathology of Assimilation, and of the Secretion of the Bile and Urine, its importance and size certainly entitled it to this consideration; but would it not have been better placed at the commencement? We advise the reader to commence with the third part, and his future studies will be materially elucidated.

